# Use of ultrasound and MRI to stage the axilla for breast cancer before and after neoadjuvant chemotherapy prior to targeted sentinel lymphadenectomy

**DOI:** 10.1007/s10549-024-07332-8

**Published:** 2024-05-03

**Authors:** Danielle Brabender, April Ballard, Sean Kim, Linda Hovanessian-Larsen, Stephen F. Sener

**Affiliations:** 1 Department of Surgery, Los Angeles General Medical Center, Clinic Tower, 6A231A, 1100 North State Street, Los Angeles, CA 90033 USA; 2 Norris Comprehensive Cancer Center and Keck School of Medicine of USC, University of Southern California, Los Angeles, CA USA; 3 Department of Radiology, Los Angeles General Medical Center, Los Angeles, CA USA

**Keywords:** Breast cancer, Axillary imaging, Neoadjuvant chemotherapy, Axillary metastasis, Sentinel lymph node

## Abstract

**Purpose:**

Prior data from this Center demonstrated that for patients who had biopsy-proven axillary metastases, were ycN0 after neoadjuvant chemotherapy (NAC), and had a wire-directed (targeted) sentinel lymphadenectomy (WD-SLND), 60% were node negative. The hypothesis of this study was that results of axillary imaging either before or after NAC would be predictive of final pathologic status after WD-SLND.

**Methods:**

For patients treated with NAC between 2015 and 2023, ultrasound and MRI images of the axilla were retrospectively reviewed by radiologists specializing in breast imaging, who were blinded to the surgical and pathology results.

**Results:**

Of 113 patients who fit the clinical criteria, 66 (58%) were ypN0 at WD-SLND and 34 (30%) had a pathologic complete response to NAC. There was no correlation between the number of abnormal lymph nodes on pre-NAC ultrasound or MRI imaging and the final pathologic status of the lymph nodes. The positive predictive value (PPV) of abnormal post-NAC axillary imaging was 48% for ultrasound and 53% for MRI. The negative predictive value (NPV) for normal post-NAC axillary imaging was 67% for ultrasound and 68% for MRI.

**Conclusion:**

The results of axillary imaging were not adequate to identify lymph nodes after NAC that were persistently pathologically node positive or those which had become pathologically node negative.

## Introduction

It was demonstrated in a prior publication from this Center that for patients with pre-treatment biopsy-proven axillary metastases who were clinically node-negative (ycN0) post-neoadjuvant chemotherapy (NAC) and had a targeted wire-directed sentinel lymphadenectomy (WD-SLND), 60% were node negative on final pathology (ypN0) [1]. For these node-negative patients, completion axillary lymph node dissection (ALND) was not performed. With median follow-up of 44 months, the axillary lymph node recurrence rate was 2%, which was consistent with other studies that showed axillary lymph node recurrence was uncommon after SLND alone. These patients would be unlikely to derive clinical benefit from completion ALND [2–8].

The finding that over half of the patients had ypN0 lymph node status on final pathology renewed interest in addressing the question as to whether axillary lymph node imaging was accurate enough to correctly identify the final axillary node status at the time of WD-SLND. If imaging could identify persistently positive axillary lymph nodes (ypN+) post-NAC, WD-SLND could potentially be omitted and ALND could be directly done. Conversely, if imaging could identify nodes that had become pathologically node-negative (ypN0), surgical axillary staging could potentially be omitted. Previous studies have demonstrated that for axillary imaging post-NAC using ultrasound, the sensitivity ranged from 49% to 87% and specificity ranged from 55% to 97% [9–15]. Specific morphologic features of axillary lymph nodes associated with malignancy have been identified, such as eccentric or concentric cortical thickness >3 mm, absent fatty hilum, rounded morphology, and increased blood flow in the thickened cortex [9–13]. The rate of malignancy in lymph nodes increased proportionally with cortical thickness, and the positive predictive value (PPV) of a replaced hilum ranged from 58% to 97% [9, 13]. Thus, previously published papers have been unable to distinguish final axillary lymph node status post-NAC based on morphologic features seen on axillary lymph node imaging.

Given the wide ranges of sensitivity and specificity for axillary ultrasound in previously published studies, this single-institution study was done to determine the accuracy of axillary imaging when consistently performed and independently re-reviewed by dedicated breast fellowship-trained radiologists. The hypothesis of the study was that the results of axillary imaging, either before or after NAC, would be associated with the final pathologic lymph node status at the time of WD-SLND.

## Methods

### Patient selection

A retrospective analysis was performed utilizing a prospective single-institution database at a large urban safety net medical center that included clinical, radiographic, and pathologic parameters of all newly diagnosed breast cancer patients who had NAC between June 2015 and June 2023. All patients in this study had biopsy-proven axillary lymph node metastases as identified by pre-treatment image-directed core biopsies. Patients without pre- and post-NAC axillary imaging (either axillary US or MRI) were excluded from the study. Patients with Stage IV or inflammatory breast cancer were not included in the analysis. The study received approval from the Health Sciences Institutional Review Board at the University of Southern California (HS: 23-03336).

### Cancer staging

Cancers were clinically staged before NAC using the 7th edition (for cases before January 2018) or the 8th edition (for cases after January 2018) of the American Joint Committee on Cancer TNM Staging System [16, 17]. The T-category was determined by the largest dimension of the tumor on clinical or radiographic examinations. Pretreatment staging scans were conducted to rule out metastatic disease for patients with ≥T3 tumors or core biopsy-proven axillary lymph node metastases.

### Axillary imaging

All breast cancer patients had routine imaging of axillary lymph nodes using ultrasound and/or MRI pre- and post-NAC, regardless of the findings on clinical examination of the axilla. Real-time ultrasound was done by radiologists specializing in breast imaging using a Canon Aplio i800 Ultrasound with an 18-MHz linear array transducer. Axillary lymph nodes were defined as abnormal on ultrasound if the lymph nodes had focal bulging of the cortex >3 mm or effacement of the hilum defined as partial (>0% to <100%) or complete (100%). Abnormal lymph nodes underwent immediate core needle biopsy followed by placement of a micro-clip. Patients’ body mass index (BMI) at time of pre-NAC imaging was used in the analysis.

MRI examinations were performed with a GE 1.5-T field scanner using a dedicated breast coil with the patient in the prone position (General Electric Healthcare, Chicago, IL). The intravenous administration of Gadavist was followed by 20 mL of saline at an injection rate of 3 mL per second using a power injector. The sequences obtained were Axial 3D T1 FS pre-contrast, Axial 3D T1 FS post-contrast (five series at 1-min intervals beginning at 60-s post-contrast), Axial T2 STIR, Axial T1, and Coronal STIR. Post-processing was performed on subtraction, MPR, and MIP images. All images were interpreted by a fellowship-trained breast radiologist using commercial software on a dedicated, independent 3D workstation with the aid of CADstream (Merge CADstream, IBM Watson Healthcare, Chicago, IL). Axillary lymph nodes were defined as abnormal on MRI if they had irregular margins, effacement of the hilum, or if there was asymmetry of the axillary lymph nodes.

For this study, ultrasound and MRI images were retrospectively independently reviewed by radiologists specializing in breast imaging, who were blinded to the surgical and pathology results. Features of lymph nodes which were evaluated included the number of abnormal lymph nodes and morphological features concerning for malignancy. The number of abnormal lymph nodes identified with ultrasound and MRI (0, 1, 2–3, >3) and the level of abnormal lymph nodes seen on MRI (level I, Levels I and II, levels I, II, and III, not applicable) were recorded. Responses to chemotherapy were categorized by either progression of disease or by the percentage of regression in abnormal lymph nodes compared to initial imaging, defined as no change (<50% regression), partial response (>50–100%), or complete response (lymph node normal post-NAC).

### Systemic treatment

All patients underwent NAC following recommendations from a weekly multidisciplinary breast conference. NAC was indicated for patients with cancer stage ≥T2, radiographically suspicious or biopsy-positive axillary lymph nodes, or those opting for breast conservation with tumors more suitable for mastectomy. The specific medications and duration of treatment were determined by the treating medical oncologists, adhering to regimens for NAC described in the National Comprehensive Cancer Network guidelines, based on the cancer stage and tumor biomarkers.

### Surgical staging

All patients had biopsy‐proven axillary lymph node metastases, received NAC, and were then ycN0 by physical examination. Wire localization of the previously biopsy‐proven metastatic lymph node containing the micro-clip was done the day before surgical staging. Periareolar injection of technetium 99m sulfur colloid and lymphoscintigram were conducted the afternoon before the operation. Methylene blue dye was utilized in the operating room if the patient did not map on the lymphoscintigram or the gamma probe failed to identify an axillary sentinel lymph node after the induction of anesthesia. A targeted wire-directed sentinel lymph node dissection (WD-SLND) was designed to remove the radioactive (“hot”) and wire-localized (“clipped”) lymph node(s). Intraoperative imaging was used to confirm the removal of the node containing the micro-clip. Excised lymph nodes were evaluated by frozen section analysis with hematoxylin and eosin staining. Patients without evidence of lymph node metastasis on frozen section analysis (ypN0) had WD-SLND alone, while those with confirmed pathologically positive nodes (ypN+) on frozen section had completion ALND. ALND was also performed for patients who did not map or in cases where the micro-clip was not retrieved during the WD-SLND.

### Final pathologic evaluation

Histopathological assessment was conducted on formalin-fixed, paraffin-embedded tissue samples. Pathological complete response (pCR) to NAC was defined as the absence of invasive cancer or ductal carcinoma in situ (DCIS) in the breast and axillary lymph nodes. The presence of lobular carcinoma in situ was not considered a pathologic diagnosis.

### Statistical analysis

Statistical analyses were conducted using SPSS^®^ v27.0 (IBM^®^ Corp, Armonk, NY, USA) and Excel v.16 (Microsoft^®^ Corporation, Redmond, WA, USA). Ultrasound and MRI images were independently assessed and compared with the pathology results obtained at the time of WD-SLND. For the evaluation of imaging findings, the following definitions were used: true-negative (TN) normal lymph nodes on axillary imaging and ypN0 on final pathology; false-negative (FN) normal lymph nodes on axillary imaging but residual cancer in lymph nodes on final pathology (ypN+); true-positive (TP) abnormal lymph nodes on axillary imaging and pathologic lymph node status ypN+; and false-positive (FP)-abnormal lymph nodes on axillary imaging but pathologic lymph node status ypN0.

Sensitivity was the percentage of patients with ypN+ lymph node status after NAC on final pathology who had abnormal axillary imaging. Specificity was the percentage of patients with ypN0 lymph node status after NAC who had negative axillary imaging. The accuracy rate was calculated as the percentage of correct test results over all test results ([TP + TN]/[TP + TN + FP + FN] × 100)%). The PPV was the percentage of patients with abnormal axillary imaging after NAC who had ypN+ lymph node status on final pathology, while the negative predictive value (NPV) was the percentage of patients with normal lymph nodes on axillary imaging after NAC who had ypN0 lymph nodes status on final pathology.

To examine the relationships between clinical factors and the rate of correct pathologic diagnosis using ultrasound/MRI, categorical variables were compared using chi-square or Fisher’s exact tests, and continuous variables were compared using Mann–Whitney *U*- or *t*-tests. *p* values < 0.05 were considered statistically significant.

## Results

Of 255 consecutive patients who fit the clinical criteria and had NAC from 2015 to 2023, 113 (44%) had pre-treatment biopsy-proven axillary metastases, were clinically node negative (ycN0) after NAC, and had a WD-SLND. At the time of the surgical procedure, 66 (58%) had ypN0 lymph node status and 34 (30%) had a pCR after chemotherapy. The N-category for the remaining 47 (42%) patients was ypN1 (1–3 positive axillary lymph nodes) in 27, ypN2 (4–9 positive axillary lymph nodes) in 15, and ypN3 (≥10 positive axillary lymph nodes) in 5. Table 1 demonstrates the response to systemic treatment for the 113 patients according to molecular subtype, tumor size, and BMI. The rates of ypN0 and pCR were significantly lower for patients with ER+HER2− cancers than for those with ER−PR−HER2− (TNBC) (*p* < 0.0001) or HER2+ cancers (*p* = 0.007), suggesting that these biomarker groups had a superior response to NAC. Of the 34 HER2+ patients, 17 (50%) had a pCR. When stratified by ER-status, 8 (36%) of 22 ER+ and 9 (75%) of 12 ER− patients had a pCR (*p* = 0.007). There was no association between tumor size or BMI stratification and the response of axillary lymph nodes to NAC.

**Table 1:** Response to neoadjuvant chemotherapy according to clinical-pathologic factors

Clinical-pathologic factors	Total *N*	ypN0 (%)	pCR (%)
ER+HER2−	50	18 (36%)	4 (8%)
TNBC	29	25 (86%)	13 (45%)
HER2+	34	23 (68%)	17 (50%)
cT1c	13	4 (31%)	3 (23%)
cT2	65	41 (63%)	25 (38%)
cT3	23	16 (70%)	4 (17%)
BMI normal (≤25.0)	30	18 (60%)	7 (23%)
BMI overweight (25.0–29.9)	29	21 (72%)	13 (45%)
BMI obese (≥30.0)	54	27 (50%)	14 (26%)
Total	113		

Table 2 illustrates the association between the number of abnormal axillary lymph nodes identified on imaging and the lymph node status on final pathology results at the time of WD-SLND. In response to NAC, there were significantly fewer abnormal lymph nodes seen on the post-NAC ultrasound or MRI than on the pre-NAC ultrasound or MRI (*p* < 0.0001). Table 3 demonstrates the performance characteristics of pre- and post-NAC axillary lymph node ultrasound/MRI imaging compared with the axillary lymph node status at the time of the WD-SLND. The probabilities that patients with pathologically positive axillary lymph node(s) at the time of WD-SLND would have abnormal node(s) on post-NAC imaging (sensitivity) were 67% for ultrasound and 61% for MRI. On the other hand, the fractions of pathologically node-negative patients having normal lymph nodes on post-NAC axillary imaging (specificity) were 48% for ultrasound and 61% for MRI. The probabilities that patients with abnormal axillary imaging post-NAC would have at least one pathologically positive lymph node at the time of WD-SLND (PPV) were 48% for ultrasound and 53% for MRI. The probabilities that patients with normal axillary imaging post-NAC would have negative lymph nodes at the time of WD-SLND (NPV) were 67% for ultrasound and 68% for MRI. The false-negative and false-positive rates for post-NAC imaging were 33% and 52% for ultrasound and were 32% and 48% for MRI. Of 35 patients having a radiographic complete response (rCR) to NAC in axillary lymph nodes, 26 (74%) were ypN0 at the time of WD-SLND. For post-NAC axillary lymph node imaging with ultrasound, the PPV for cortical thickening was 47% and for lymph node effacement was 48%. For post-NAC imaging with MRI, the PPV for asymmetry in lymph nodes was 54%.

**Table 2:** Association between the number of abnormal axillary lymph nodes identified on imaging and the final pathology results

Number of abnormal nodes			Final pathology results		
	ypN0	ypN1	ypN2	ypN3	Total
*Pre-NAC*					
Ultrasound					
0	3	0	0	0	3
1	27	16	6	0	49
2–3	28	6	8	5	47
>3	3	2	0	1	6
MRI					
0	7	1	0	0	8
1	10	9	0	1	20
2–3	17	9	8	0	34
>3	13	3	2	3	21
*Post-NAC*					
Ultrasound					
0	28	7	3	4	42
1	27	14	8	1	50
2–3	3	1	3	1	8
>3	0	0	0	0	0
MRI					
0	28	5	5	3	41
1	12	8	1	1	22
2–3	6	3	6	1	16
>3	0	0	0	0	0

**Table 3:** Performance characteristics of axillary lymph node imaging before and after neoadjuvant chemotherapy based on node status at WD-SLND

Axillary lymph node imaging	True positive (*N*)	True negative (*N*)	False positive (*N*)	False negative (*N*)	Total (*N*)	Sensitivity (%)	Specificity (%)	Positive predictive value (%)	Negative predictive value (%)
*Pre-NAC*									
US	44	3	58	0	105	100	5	43	100
MRI	35	7	40	1	83	97	15	47	88
*Post-NAC*									
US	28	28	30	14	100	67	48	48	67
MRI	20	28	18	13	79	61	61	53	68

As demonstrated in Table 4, for post-NAC axillary ultrasound the PPV for TNBC was 13%, significantly lower than for ER+HER2− (*p* < 0.001) or HER2+ (*p* = 0.028) cancers. The NPV for ER+HER2− cancer was 44%, significantly lower than for TNBC (*p* = 0.022) or HER2+ (*p* = 0.022) cancers. Tumor size and BMI stratification were not associated with the predictive value of post-NAC axillary ultrasound imaging.

**Table 4:** Performance characteristics of post-NAC axillary lymph node ultrasound according to molecular subtype of cancer based on axillary lymph node status

Clinical-pathologic factors	Total US	True positive (*N*)	True negative (*N*)	False positive (*N*)	False negative (*N*)	Positive predictive value (%)	Negative predictive value (%)
ER+HER2−	41	17	8	6	10	74	44
TNBC	28	2	10	14	2	13	83
HER2+	31	9	10	10	2	47	83
Total	110	28	28	30	14		

## Discussion

This study was done in breast cancer patients with pre-treatment biopsy-proven axillary lymph node metastases who had axillary imaging pre- and post-NAC. Despite axillary lymph node imaging with ultrasound and MRI demonstrating a decrease in the number of abnormal lymph nodes after chemotherapy, imaging was not adequate to correctly identify the axillary lymph node status at the time of sentinel lymphadenectomy. The false-negative rates for post-NAC imaging were approximately 30% for ultrasound and MRI, and the false-positive rates were approximately 50% for ultrasound and MRI, considerably below levels at which imaging might be an acceptable substitute for surgical staging of axillary lymph nodes.

The findings of this study confirmed those of four previously published studies, in which the diagnostic performance of axillary imaging was similarly not adequate to substitute for surgical staging of the axilla post-NAC (Table 5) [10–12, 15]. Of the 3212 patients presented in Table 5, including the 113 from the current series, 1378 (43%) had ypN0 lymph node status at the time of surgery. In aggregate, the median sensitivity, specificity, PPV, and NPV for post-NAC US were 67%, 69%, 77%, and 57% and for post-NAC MRI were 61%, 60%, 77%, and 51%. In three of the five studies, the radiographic images were not re-reviewed by a small number of breast-trained radiologists, potentially increasing the possibility of interobserver variation [10–12, 15]. Also, in none of the previous studies was a micro-clip placed at the time of core needle biopsy of the abnormal axillary lymph node identified by imaging. In the current series, all patients had pre-treatment evaluation of the axilla by clinical and radiological examination and core biopsy of abnormal lymph nodes with placement of a micro-clip at the time of biopsy. Wire localization of the biopsy‐proven metastatic lymph node containing the micro-clip was done, and targeted wire-directed sentinel lymph node dissection (WD-SLND) was designed to remove the radioactive (“hot”) and wire-localized (“clipped”) lymph node(s). Intraoperative imaging was used to confirm the removal of the node containing the micro-clip. Using this process has been shown to reduce the risk of leaving positive lymph nodes in the axilla (false-negative rate) at the time of SLND, thereby allowing for a more accurate assessment of the pathologic status of the axillary lymph nodes at the time of surgery [18]. Axillary ultrasound without the use of wire-directed SLND was also demonstrated in American College of Surgeons Oncology Group Z1071 trial to lower the false-negative rate for SLND when at least two sentinel lymph nodes were removed [19].

**Table 5:** Performance characteristics for ultrasound and MRI in selected previous publications, comparing imaging results with final pathologic status of axillary lymph nodes

Author (year) Ref. #	Number of patients	Images re-reviewed	Included cN0 patients^a^	Sensitivity (%)		Specificity (%)	PPV (%)	NPV (%)
Samiei (2021) [15] *Meta-analysis*	2380	No	No	*Post-NAC*				
				US	65	69	77	50
				MRI	60	76	78	58
You (2015) [11]	139	No	No	*Post-NAC*				
				US	50	77	84	38
				MRI	72	54	80	44
Heiken (2013) [12]	272	Yes	Yes	*Post-NAC*				
				US	70	58	71	57
				MRI	61	59	75	43
Peppe (2017) [10]	308	No	No	*Post-NAC*				
				US	71	88	83	79
Current Series	113	Yes	No	*Pre-NAC*				
				US	100	5	43	100
				MRI	97	15	47	88
				*Post-NAC*				
				US	67	48	48	67
				MRI	61	61	53	68

^a^cN0 defined as node-negative pre-NAC by clinical exam and tissue sampling using fine-needle aspiration or core needle biopsy

Similar conclusions to those in the current study were reached by Barrio et al. using a different study design in which only pre-NAC images were compared with the pathologic status of axillary lymph nodes [20]. In that study of 402 patients treated from 2008 to 2016, pre-NAC imaging was done at the discretion of the treating physician(s). The cN0 designation was defined by clinical examination of the axilla, and information regarding axillary imaging was obtained by chart review. Of 402 cN0 patients, 111 (28%) had an ypN+ axillary lymph node status. Of patients with abnormal lymph nodes on pre-NAC axillary imaging, 54% were ypN0 post-NAC. Conversely, 20% of patients with normal lymph nodes on pre-NAC axillary imaging were ypN+ post-NAC. The authors concluded that pre-NAC axillary imaging did not accurately predict the need for axillary surgery post-NAC.

Recent advancements in the predictive capability of axillary imaging have utilized radiomics and deep learning or artificial intelligence (AI)-based integration of imaging and clinical-pathologic information. For example, in a recent study three publicly available image recognition tools that leverage convolutional neural networks to develop a machine learning algorithm were used to detect axillary lymph node metastases on US [21]. The AI programs were initially trained on an internal imaging data set of 756 patients, and when a test set of clinically node-negative patients was evaluated, the deep learning model was more effective than a radiologist at identifying pathologic axillary lymph node metastases (AUC 0.89, sensitivity 85%, specificity 73%, PPV 76%, NPV 81%) [21]. Other studies utilizing deep learning programs with similar results have been published, but the use of proprietary software or algorithms has limited generalizability of results and accessibility to other investigators [22, 23]. The utilization of AI with imaging for the detection of breast cancer is also a rapidly progressing field, and several FDA-approved programs are available for this purpose, but there are no currently available AI products for evaluation of the axilla [24].

An important strength of this study was its performance at an urban safety net medical center. The findings confirmed the feasibility and efficacy of performing sophisticated treatment protocols in a safety net medical center. The original data produced in clinical trials using NAC were generated at large academic centers. This study may support the generalizability of conclusions from prospective randomized trials and retrospective studies in which Hispanic patients have historically been underrepresented. In addition, patients who were uninsured or had Medi‐Cal or Medicaid expansion healthcare plans were able to obtain access to state‐of‐the‐art breast cancer care.

This study had several potential limitations. Although consistency of standardized imaging techniques and re-interpretation of images by two breast radiologists were strengths of this study, the retrospective nature of the study design presented the potential for observer bias because production of the ultrasound images was operator dependent and the image reviews were based on subjective assessment. Also, chemotherapy and radiotherapy regimens were standardized, but they differed by individual patient and were not controlled in data analysis.

## Conclusion

The hypothesis of the study, that the results of axillary imaging would be associated with the final pathologic lymph node status at the time of WD-SLND, was not confirmed. Even with expert radiologists and consistent radiographic methodology, axillary imaging was not adequate to replace surgical staging of axillary lymph nodes.

## Data Availability

Data used in this study are not publicly available but are available from the corresponding author upon reasonable request.
